# FINDING FORTITUDE: SOCIAL CONVOYS OVER THE LONG HAUL

**DOI:** 10.1093/geroni/igae098.1162

**Published:** 2024-12-31

**Authors:** Rita Hu, Kristine Ajrouch, Elaine Wethington

**Affiliations:** Chair; Co-Chair; Discussant

## Abstract

As populations globally face the opportunities and challenges of aging, social convoys across the lifespan are pivotal in navigating the complexities of later life. This symposium explores the dynamic interplay between social relations and aging, emphasizing the protective and predictive nature of social networks over the lifespan and in different cultural contexts. Becheroui and colleagues explore familial care in Lebanon across age groups, focusing on the balance between cultural expectations and the need for policy support. Antonucci and colleagues examine how socioeconomic status affects changes in health through social relations, particularly the influence of parent-child dynamics among men and women. Hu and colleagues also provide a longitudinal perspective, showing how social network trajectories over nearly three decades influence later-life cognitive health, emphasizing the enduring importance of weaker ties for cognitive health later in life. Webster and colleagues present evidence of how educational attainment within one’s social network at one point in time contributes to cognitive health later in life, pointing to the broader implications of social environments on cognitive well-being. Aimed at fostering a comprehensive understanding of social convoys in the aging process, this symposium invites a cross-disciplinary dialogue to enhance the support for aging individuals worldwide, embodying the symposium’s theme of finding fortitude through the power of social relationships.

